# Creating patients: how technology and measurement approaches are misused in diagnosis and convert healthy individuals into TMD patients

**DOI:** 10.3389/fdmed.2023.1183327

**Published:** 2023-10-12

**Authors:** Charles Greene, Daniele Manfredini, Richard Ohrbach

**Affiliations:** ^1^ Department of Orthodontics, College of Dentistry, University of Illinois at Chicago, Chicago, IL, United States; ^2^ Orofacial Pain Unit, School of Dentistry, Department of Biomedical Technologies, University of Siena, Siena, Italy; ^3^ Oral Diagnostic Sciences, University at Buffalo School of Dental Medicine, Buffalo, NY, United States

**Keywords:** temporomandibular disorders, diagnosis, technology, device, instrument, validity

## Abstract

The advances made in recent years regarding technological approaches to medical and dental diagnosis are impressive. However, while those tools, procedures, and instruments may produce an improved clinical diagnosis or discover a new disorder, they also can be misused and misinterpreted in various ways. In the field of temporomandibular disorders (TMDs), the very nature of those conditions is similar to common orthopedic problems elsewhere in the body. Yet, beyond imaging of the affected areas, there have been few important new technological approaches to augment the traditional history and examination for a sufficient diagnosis of such problems. The traditional approach is exemplified by the Diagnostic Criteria for Temporomandibular Disorders, which has high inter-examiner reliability and diagnostic validity; translations into over 20 languages allow for widespread use. In contrast and unfortunately, the TMD field is replete with a variety of so-called diagnostic instruments and procedures, which have not been tested for diagnostic validity; these instruments and procedures, through misuse, are capable of complicating a true diagnosis of patients who present with symptoms, while also creating new patients by finding so-called abnormalities in healthy subjects. This paper discusses those technological approaches and their misuse with respect to TMD diagnosis from a critical viewpoint, and the authors argue that there are significant risks for patients if their uncritical implementation becomes accepted and widespread. Therefore, dentists are encouraged to reject the proposed application of such technological approaches to diagnosis of the stomatognathic system.

## 1. Introduction

Although based on fundamental mechanisms of interoception (i.e., the sense of one's own self) (1) people vary in their self-detection of medical problems, with some individuals reporting alterations in bodily processes before any doctor or diagnostic instrument could possibly detect change, whereas other individuals may ignore warning signals until very late in a disease process. Consequently, respect for patient autonomy drives a major theme: the patient's “complaint” is central to how any disease process should be appraised by the health care provider, yet the examiner must be vigilant for new onset disease processes. Within this framework, the classical approach utilizes a symptom history and a physical examination; further tests are ordered only based on the principle of effective prescription, viz., only when the outcomes of such tests are expected to impact the diagnostic hypothesis. The latter point is underscored by observations of expert clinicians and their decision-making for when to order tests, and how infrequently tests actually change the diagnosis (2). These principles for standard clinical practice evolved in recognition that health and disease reside within a complex continuum. Therefore, we must protect patients from both false-negatives resulting from inadequate history or examination, and false-positives resulting from over-zealous evaluations.

An example of good clinical practice, which illustrates that technology is not necessarily required for highly valid clinical assessment, is the Diagnostic Criteria for Temporomandibular Disorders (DC/TMD), which relies solely on symptoms from initial self-report and from provocation during a clinical examination. Sensitivity and specificity for pain-related diagnoses of myalgia, arthralgia, and headache attributed to TMD range 89%–90% and 87%–99%, respectively (3). Reliability across multiple examiners for pain-related diagnoses can range 0.90–1.0, depending on the sample mixture of patients with TMDs (4). Of note, the pain-related TMDs are the most prevalent among the TMDs, and as such the clinical need matches the strengths of the DC/TMD. Equally telling, perhaps, is that the diagnostic sensitivity using the clinical procedures within the DC/TMD for most disc displacements and degenerative joint disease is poor to fair (range 34%–55%), the authors explicitly state that this is a screening level assessment, and they direct the user to also investigate the presumed joint condition with appropriate imaging when the patient has significant mechanical problems, when significant disease is suspected, or when prior treatment of the joint was unsuccessful (3). The DC/TMD represents a clear alternative to these non-evidence based clinical practices. Because the DC/TMD has high reliability and validity, it does not create diseases disproportionate or unrelated to a chief complaint, and it does not result in harm or potential harm to the patient.

Like all medical fields, dentistry provides examples of situations in which a full clinical assessment may uncover important findings that are not part of the patient's complaints; these findings range from problems associated with teeth or periodontal tissues as identified by both a thorough clinical exam as well as routine imaging, to more complex diseases such as oral cancers. The detection of such problems is intrinsic to the expected standard of dental practice.

In contrast, it is unfortunate that certain diagnostic approaches to the field of temporomandibular disorders (TMDs) produce outcomes in which the chief complaint does not play a central role. Instead, these diagnostic approaches are based primarily on various types of testing used to identify so-called abnormalities that should not require clinical correction. A series of review papers published by the University of Buffalo group in the 1990s made it clear that there was no existing evidence to support the use of various electronic diagnostic machines and instruments (5–7). While those reviews, published more than 30 years ago, directly identified the need for advocates of such diagnostic devices to engage in solid science, claims regarding yet other diagnostic instruments and procedures continue to emerge and continue to lack supporting evidence. Subsequent reviews tell the same story (8–12). Medical devices are certainly not exempt from the appeal for being impressive-looking, and the possession of such devices in clinical settings is clearly fashionable; in contrast, diagnostic validity research is difficult to conduct and therefore neither appealing nor fashionable. In short, there are no benefits yet demonstrated for the clinical use of such devices, and any advancements are anchored in misplaced precision. Yet, without evidence of validity, there can be no utility. Two scenarios are associated with such testing.

First, it is common to see a variety of inappropriate or unnecessary technologies and measurements being utilized during the diagnostic process for patients who have TMDs. These strategies usually magnify the problems and lead to a proposed treatment protocol that results in irreversible changes to the dental occlusion, mandibular position, or both as part of the treatment strategy to “correct” the so-called abnormality. Second, the same variety of technologies and measurements are utilized on asymptomatic individuals presenting to the dental office for routine checkups, thereby creating non-existing diseases and treatment needs. In both scenarios, there is an obvious possibility of either magnifying or creating a serious iatrogenic disease when the patient may actually have either a simple condition or no disease at all. Third, appropriate technologies and measurements can also be misused; inadequate training in relevant information integration can result in this type of problem. For example, an MRI can identify the anterior band of a TMJ disc as being anterior to its expected location the MRI cannot interpret this as a common condition that has no clinical relevance in the absence of pain or mechanical limitations. Yet, the MRI report can be misused clinically to inform the patient regarding the presence of a diseased disc.

## 2. Temporomandibular disorders and their instrumental magnification

The present paper focuses on TMDs and addresses clinical practices that do not align with the above core observations regarding a patient-centered approach to human disease and illness. TMDs are a group of musculoskeletal disorders primarily characterized by regional pain and mechanical disturbances associated with the temporomandibular joint (TMJs). The common TMDs include muscle pain disorders, TMJ disc disorders, and TMJ degenerative joint disease (3). Whereas the combined prevalence of these disorders when chronic is approximately 10%, (13) the annual incidence of first life-time onset of painful TMDs is approximately 4% (14). Furthermore, as shown by the well-known OPPERA (Orofacial Pain: Prospective Evaluation and Risk Assessment) studies, extensive research regarding risk factors for this group indicates that a TMD condition appears more frequently in persons with other pre-existing health problems, including musculoskeletal symptoms elsewhere in the body, psychological distress, and other painful conditions (e.g., fibromyalgia, irritable bowel syndrome, headaches) (15). The initial onset of a painful TMD reflects multiple system dysregulation, and with the transition to chronicity, those dysregulated systems continue to exert influence and other forms of dysregulation contribute as well (16). Only about 10%–20% of those with chronic painful TMDs are without any other pain disorders (17). Furthermore, the evidence clearly indicates that the severity of co-morbid conditions worsens with time (18) and pain becomes centralized (19). Consequently, for these disorders, attempts to focus diagnosis and treatment solely at the correction of the masticatory system may be misguided at best, and iatrogenic at worst (15).

While less is known regarding the causes of TMJ disc conditions, (20) they are highly prevalent (about one third of individuals) in the general population. The vast majority of those individuals report no or minimal symptoms and no relevant impact on function (21). The disc position itself has been shown to be highly variable in both normal individuals as well as in persons with TMDs (22). Despite multiple papers over the past two decades refuting the etiologic theories that blame the dental occlusion for causing TMDs, (23–31) beliefs regarding the presumed causal role of malocclusion or other structural aberrations persist within the dental profession. Unfortunately, those beliefs usually lead to rejection of the available evidence supporting the relevance of the biopsychosocial model for TMDs (28). Consequently, clinicians may be persuaded to use approaches that focus on anatomical relationships, structural “abnormalities”, or minor disease findings that are biologically unimportant. Equally critical, the focus on technological and measurement approaches leads to an inadequate understanding of the patients, their pain, and the significance of central mechanisms. With such a one-sided perspective, the nice-looking numbers that technology can produce become the clinical evidence for a purportedly significant TMD problem—despite the absence of any disease process. We now address (i) the conceptual problems underlying the validity of those instrumental approaches, (ii) the kinds of approaches that are used, and (iii) the necessary path forward.

## 3. The problems of false analogies

A common rationale offered by proponents of technological approaches to the diagnosis or discovery of TMDs is to compare the medical and dental fields. They argue that many medical conditions are discovered by a variety of modern technologies, and also that many clinical diagnoses require a series of follow-up testing procedures. Examples may include: cardiovascular conditions (e.g., ECG, ultrasound, angiography, electrophysiology); organ diseases (e.g., liver or kidney function tests, colonoscopy); brain pathology (e.g., MRI, MRA, PET scan); and connective tissue diseases (e.g., blood analysis, serology, immune system testing).

The implication is that failure to adopt various modern technologies in the field of TMDs is contrary to modern health practices, and therefore the dental profession must move forward accordingly. A claim is often made that important sub-clinical states (that is, without symptoms or signs) exist and must be detected early, and therefore technology-enhanced assessment of the body will discover an underlying disease. As an example of the futility of such claims, sub-clinical pain similar to that of TMDs occurs frequently in the population and is self-limiting, and such occurrences have no appreciable predictive power for subsequent onset of clinically important disorders (32). Accordingly, it is notable that the majority of orthopedic disorders do not routinely receive evaluations using any specific technology beyond imaging. Physicians do not routinely perform screening tests for joint disorders by imaging or measurement procedures, nor do they routinely use electromyography (EMG) to examine the musculature surrounding every joint. Instead, they recognize that most orthopedic problems are reported, not “discovered”, which is exactly how things should be in our field. Therefore, the various modalities and procedures described in the following sections can generally be considered as inappropriate due to their misuse. Their high risk for false-positive findings—which generally occur in the absence of any symptoms—is a clear warning against their use. For example, a patient with myofascial pain may have changes in the position, shape or size of the TMJ condyles, but such findings are common in the random population as well. Similarly, a patient who presents for dental checkup may have TMJ clicking, or deviation upon mouth opening, or tenderness to palpation of certain muscles—but these do not suggest or predict a diagnosis of any specific TMD. “The variability of these kinds of ‘findings’ in the normal population guarantees that some people may be opening their mouth in a crooked path, while others may be hypersensitive to having a doctor push on their muscles; these are not pathologic findings, and should not be overinterpreted in the absence of other clinical criteria for establishing a TMD diagnosis.”

## 4. Problems affecting the validity of instrumental approaches

### 4.1. Defining normal

Recognition of normal variation in biology and its expression in the physical phenotype of the individual is central to how the psychophysical status of any individual patient is interpreted, including the distinction between variation in normal vs. disease that may be benign.

For example, blood pressure may be pathologic, or it may be benign; if benign, then is it “normal”? Normality of any measured process, such as blood pressure, is determined by community standards within science and medicine, and utilizes appropriate statistical parameters such as central tendency, dispersion, range, and distributional shape. As such, the blood pressure device cannot answer that question of whether an observed blood pressure is “normal”; family history and current aerobic functioning might provide answers. At the pathology level, biological changes occur across a wide spectrum of severity and across a wide spectrum of time, with differing implications for whether (or when) a change represents a disease process; “sub-clinical” describes one possible state well. However, the majority of instrumental approaches to TMDs are deficient in terms of adequately defining normal; in fact, some approaches essentially classify nearly everyone as abnormal. For example, a maximal mouth opening at 40 mm (as based on the use of a simple ruler) is often used as the “normal” cut-off for range of motion (33). However, some patients may have a lifelong limitation in opening, as compared to a statistical definition of normal; therefore, neither the ruler nor a tracking device that measures range of motion with 0.1 mm precision can determine what is normal. A common scenario regarding what is normal occurs daily in clinical dentistry and revolves around occlusion. A perfect occlusion is relatively rare—say, 5% of the population—yet a normal morphological occlusion need not be perfect (34).

The problem in defining normal is not a technical one, but rather is intrinsic to biological systems and which must be contextualized for age, sex, previous medical history, and many other variables: they adapt to change. Tissue change may be evident to the patient, but perhaps without any biological impairment; one function of the body is to adapt, and adaptations may be more successful than any known therapy. Similarly, an impairment need not necessarily be accompanied by limited function; for example, decreased hearing (an impairment) may be compensated by adequate visual skills in understanding speech. Finally, quality of life is heavily influenced by all of the known psychosocial factors that influence health overall. In summary, how pathological change in the body impacts on the individual is influenced by biological and behavioral adaptation, and these forces must be considered in any definition of clinical “normal” vs. “abnormal” (35).

### 4.2. Problems of circularity

Circularity can occur between how normal is defined and the establishment of a cut-off without statistical or biologic evidence of its validity. For example, it has been claimed that the condyle should be in the “middle of the fossa” on a radiographic image. However, many studies have shown that condyles are distributed in various relationships to their fossae in both individuals with a TMD as well as individuals without a TMD. Therefore, one cannot use this incorrect assumption to create a diagnosis of condylar displacement and plan extensive treatments to reposition the condyle—a prime example of circular reasoning. Similar problems of circularity exist in how normal is defined for all of the instrumental approaches that classify an individual as having a TMD.

## 5. Imaging: why pictures may depict anatomic or pathologic findings that are not clinically significant

Tremendous progress has been made in recent years in the technologies associated with medical imaging. Not all of these are applicable to conditions managed by the dental profession, but three are: (1) panoramic radiographs; (2) cone beam computed tomography (CBCT); and (3) magnetic resonance imaging (MRI). All three of these provide images of the TMJs, with the first two showing only hard tissues and the last showing soft tissues. The important clinical questions are: What is the appropriate usage for each of these modalities, and how should the images produced be interpreted?

Many dental offices have been using panoramic films for decades, while CBCT has only recently become more widely used; MRIs of course must be performed in dedicated imaging centers. While the main purpose of using those office imaging modalities as part of screening is to examine the dentition and surrounding structures, some practitioners focus on the TMJ structures and reach various conclusions. For example, two-dimensional panoramic films might show significant morphologic differences between two condyles, but this is not an uncommon finding since bilateral TMJs are only rarely symmetrical (36). The three-dimensional CBCT can be manipulated on a computer to better look at those same variables, and in addition spatial relationships between condyles and fossae may be analyzed.

But in the absence of symptoms, what is the significance of finding asymmetric morphology or imperfections of the cortical bone on top of a condyle? If the condyle appears to be located in the fossa in a so-called posterior position, how should that be interpreted, and what should be done about it? These are the kinds of questions that only a longitudinal prospective study could answer. The same questions can be asked even if the patient has symptoms of myogenous or arthrogenous jaw pain. If the patient has a clicking joint (with or without pain), should an MRI be obtained routinely? [Published guidelines would say no (33)]. Since an MRI most likely would show an anteriorly displaced disc (ADD), what kind of clinical management would be indicated in the absence of symptoms specific to the disc position?

Unfortunately, the answer to these questions is all too often oriented toward some kind of clinical intervention. Patients with a myogenous pain problem may be erroneously re-classified as having degenerative joint disease (DJD; osteoarthritis), and their treatment protocols are amended accordingly. Patients with a displaced disc are told this can be a significant future problem, and some type of recapturing procedure with oral appliances or discopexy surgery is recommended. In children and adolescents, it has been reported that a mandibular growth asymmetry will occur if there is a unilateral ADD, and orthodontists have been told they must correct the disc problems before starting treatment.

However, the dental literature on these topics does not support many of those opinions. With one-third of the population having some type of ADD (mostly asymptomatic), a small percentage may have minor progressions such as painful clicking or limited opening requiring conservative management, while only a few individuals may develop a serious problem of pain and dysfunction requiring a major intervention, which should not aim at restoring dental occlusion anyway (37). Orthodontists can usually compensate for the slight asymmetries they encounter, so it is hard to defend disc recapturing procedures in all their young clicking patients. Finally, the position of the condyle relative to the fossa as well as the finding of peculiar morphology is neither diagnostic nor predictive of any particular future problems; instead, it is generally attributable to normal variations of these structures. Therefore, TMJ imaging technologies must be interpreted with caution, and the findings frequently do not require any clinical reaction.

## 6. Major problems with technological diagnostic approaches to TMD diagnosis

For decades, the TMD field has been permeated with proposals of purported diagnostic instruments. An inevitably incomplete list of gadgets that are available on the market includes surface electromyography (sEMG), kinesiography and jaw tracking devices, axiography and other tools for condylar tracking, joint vibration analysis, occlusal contacts evaluation, and postural platforms. We do not describe these in detail, in that the proponents of such instruments and methods already provide easily found glowing accounts via the internet of what the instruments and methods purportedly do. However, a critical read of those accounts quickly highlights the purely anecdotal nature of the claims. Generally, the only kind of “evidence” that such websites provide (and again, we are not listing these: the interested reader can easily find them) is under the general header of “Testimonials”, as though beliefs in diagnostic efficacy are sufficient. Neither examples nor details provided here would affect the fundamental problem: use of technology without evidence is not good clinical practice.

The usefulness of those devices in the diagnostic process should be evaluated according to proper evidence supporting or refuting their validity (38). A critical review of their validity goes beyond the scope of this paper, but the available evidence from over 30 years ago (5, 7) as well as more recently published (9) indicates consistently that all such devices lack the necessary effectiveness for diagnostic purposes. A claim frequently made for those devices is that they have technical validity (e.g., sEMG may accurately measure muscle fiber contraction), and therefore they can be used for diagnosis. Devices can be misused, despite technical validity. Moreover, diagnostic validity has separate requirements, (38) and technical validity is not a substitute.

Negative findings as far as the correlation with clinical symptoms have been reported for sEMG, jaw tracking devices, and postural platforms, viz., findings with those instruments are often not repeatable, (39–41). Also, such instruments may produce findings that are too similar between patients and non-patients, (10, 42, 43) or they are not useful to monitor patient changes over time (44). Jaw and condylar tracking devices do not show acceptable correlation with TMJ imaging findings (11, 45, 46); therefore, concepts about the need to evaluate the condylar position or guidance and to “deprogram” the jaw muscles as part of the TMD treatment plan have been abandoned (47, 48). At the expert level, consensus recommendations currently state that TMD diagnosis should result from a combination of history taking, clinical assessment, and, in some selected cases, imaging (33, 49, 50).

The negative impact of purported diagnostic instruments in TMD practice unfortunately goes far beyond their lack of validity. Indeed, all the above instruments are still associated with treatment protocols based on the old Phase I-Phase II paradigm, viz., the purported abnormality detected with the device should be treated with approaches based on the correction of mandible position and dental occlusion. Despite the lack of support as repeatedly highlighted in previous papers, (51–53) the use of such instruments remains fascinating for many practitioners, under the promise that they can provide so-called objective findings that lead to more sophisticated TMD treatment procedures. Unfortunately, findings that are potentially technically reliable but diagnostically invalid and lack a biological basis are without value and are also potentially damaging to the patient. Paradoxically, the use of fancy instruments is attractive also for the patients, who may believe in technological doctors more than in caregivers who focus their activity on traditional evidence-based concepts and recommendations. The continuing anecdotal tradition within clinical dentistry, when encountering masticatory system function and pain or other symptom complaints, wherein testimonials and patient-series are considered adequate evidence is a serious problem. As the Oxford evidence hierarchy indicates, testimonials and case-series represent the lowest form of evidence and should not be relied upon, especially when better evidence is available (38, 54).

## 7. The creation of new patients

In addition to the misuse of diagnostic tools in the TMD field, the use of those instruments is often recommended to plan routine dental treatments (e.g., orthodontic alignments or prosthodontic restorations) based on a purported goal of ideal TMJ function. Healthy individuals are prescribed oral appliances to “normalize” condylar trajectory of movement or to “deprogram” muscles, while extensive prosthodontic treatments are “justified” to put the condyle in a certain position, and headaches are “diagnosed” with postural platforms that apparently show the need for dental correction. All of this is currently advertised under the umbrella banner term of “functional dentistry”, which includes several procedures that require extensive treatments with the goal of normalizing some purported abnormal instrumental findings of the TMJ or correcting so-called “secondary malocclusions”. As a result, parameters such as the condylar trajectory, the evaluation of hinge axis, the analysis of occlusal plane, and the chair-side EMG assessment of muscle function, have been arbitrarily introduced into the daily practice of some prosthodontic and orthodontic communities as being diagnostic. The practitioners who accept and utilize the “findings” from these modalities can then justify treatments that go far beyond the complaint of symptomatology. Browsing the internet, the social networks, and the many related advertisement posts, it seems clear that the introduction of instruments into the daily practice has led to the creation of new, non-existing diseases and new patients. Both mis-treatment and over-treatment are a consequence.

## 8. Conclusions

This paper has pointed out how the misuse in a clinical setting of a variety of technological diagnostic procedures and electronic instruments carry the twofold risk of exacerbating TMDs in symptomatic individuals, as well as creating non-existing diseases in healthy individuals.

## Data Availability

The original contributions presented in the study are included in the article/Supplementary Material, further inquiries can be directed to the corresponding author.

## Author note

CG is Clinical Professor Emeritus at the University of Illinois at Chicago.
